# Factor XIII Deficiency Associated With Noonan Syndrome

**DOI:** 10.7759/cureus.14150

**Published:** 2021-03-27

**Authors:** Zeni Kharel, Anjan Katel, Arun Neupane, Pradumna Panday, Madan Aryal

**Affiliations:** 1 Internal Medicine, Rochester General Hospital, Rochester, USA; 2 Medicine, Kathmandu University School of Medical Sciences, Dhulikhel, NPL; 3 Nursing and Critical Care, Alta Bates Summit Medical Center, California, USA; 4 Medicine, Enloe Medical Center, Enloe Regional Cancer Center, California, USA

**Keywords:** noonan syndrome, factor xiii deficiency, bleeding

## Abstract

Noonan syndrome (NS) is an autosomal dominant disorder with multisystem involvement. NS can be associated with bleeding disorders due to defects in platelet function or coagulation factors and diagnosis can be challenging. Factor XIII (FXIII) deficiency is uncommon in patients with NS. We present a case of NS who presented with bleeding in both thighs and was diagnosed to have deficiency in FXIII.

## Introduction

Noonan syndrome (NS) is an autosomal dominant disorder with incidence of around 1 in 1,000-2,500 individuals [1]. Multisystem involvement and phenotypic variations are common in NS and include a wide range of abnormalities such as skeletal and thoracic abnormalities, developmental delay, congenital cardiac anomalies, intellectual disabilities, and short stature. Hematological disorders are seen in 30%-72% of patients [1] and bleeding is the most common hematological disorder [2]. Bleeding in NS is thought to be related to dysfunction of platelets, thrombocytopenia, von Willebrand disease (VWD), and deficiencies of clotting factors occurring in isolation or in combination [3]. Factor XI deficiency and platelet dysfunction have been described most frequently [4-5]. We report Factor XIII (FXIII) deficiency associated with NS which has only been reported once before [1].

## Case presentation

A 27-year-old man with NS presented with progressively worsening ecchymoses involving the anterior aspect of both thighs for four days. The ecchymoses was associated with pain, difficulty with ambulation, and fatigue. He was initially seen at another hospital for the same and was transferred to our center due to sudden drop of hemoglobin by over three points in one day. He denied any history of trauma or known bleeding history. His presenting vital signs included blood pressure 121/76 mmHg in the left upper arm, heart rate 116 beats/min, and respiratory rate 16 breaths/min. He had generalized pallor and ecchymoses involving the anterior aspect of bilateral thighs. He had short stature and low set ears but otherwise rest of the exam was normal.

**Figure 1:**
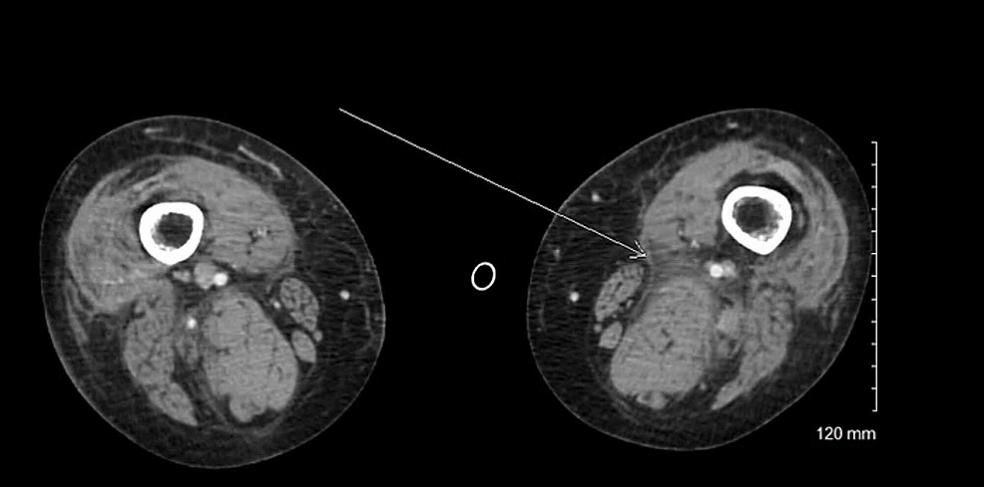
CT scan of bilateral thighs showing subcutaneous tissue edema within the deep muscular compartments.

On investigation, his hemoglobin was 7.4 g/dL, reticulocyte count 9.6%, white blood cell 11,200/mm3, and platelet count 119,000/mm3. The liver function tests [aspartate aminotransferase (AST), alanine transaminase (ALT), alkaline phosphatase (ALP), and bilirubin] and renal function tests (blood urea nitrogen, creatinine) were within normal limits. The electrolytes including sodium, potassium, calcium, magnesium were also within normal limits. CT (Figure 1) of the thighs showed subcutaneous tissue edema within the deep muscular compartments. CT angiography of abdominal aorta and lower extremities revealed bleeding in bilateral anterior thighs. Coagulation profile including prothrombin time (PT)/INR, partial thromboplastin time (PTT), fibrinogen, von Willebrand panel, and platelet function assay was unremarkable. Hemoglobin electrophoresis, lactate dehydrogenase, haptoglobin, vitamin B12 level, folate level, iron panel (iron saturation, ferritin, transferrin, and iron binding capacity) and peripheral blood flow cytometry all returned normal. Further testing to establish the cause of ecchymosis included Factors V, VII, VIII, IX, X, XI, XII, and XIII activities. Given that factor activity level was unavailable at the time of patient’s presentation, he was empirically treated with fresh frozen plasma (FFP) (400 mL at the time of presentation and 200 mL the following day). His hemoglobin and hemodynamics were closely monitored. His ecchymoses significantly improved after three days with improvement in the ability to ambulate. He was discharged and advised outpatient follow-up. He was seen in the clinic the following week and found to have normal factors V, VII, VIII, IX, X, XI, and XII activities but FXIII activity was found to be low at 31% (reference range: 69%-143%). Three months after the initial episode, he continued to have improvement in the ecchymoses without any recurrence. FXIII activity level measured at the three-month visit was 62%. He has been managed expectantly with plan to give cryoprecipitate and antifibrinolytics (for mucosal bleeding) and FXIII replacement for severe or major organ bleed.

## Discussion

We describe a case of NS presenting with bilateral thigh bleeding but normal PT, PTT, platelet, and fibrinogen levels. Given unknown cause of bleeding, our patient was empirically treated with FFP.

Although NS is an autosomal dominant disease, it can also occur due to sporadic or de-novo mutations. Genetic mutations are present in around 60% of patients. The most commonly involved mutations are in PTPN11 (50%), SOS1 (15%), KRAS (<5%), and RAF (3%-17%). NS is characterized by heterozygous phenotypic manifestations ranging from mild to severe. The most consistent clinical features are low set ears, hypertelorism, short stature, and pulmonic stenosis. Prognosis of NS depends on the severity of cardiac defect; however, many patients lead an average life span with minimal morbidity [6].

In a systematic review published in 2018 analyzing bleeding disorder in NS using data published between 1965 and 2014, 90% patients had some laboratory abnormality (coagulation or platelet function abnormality). Factor XI deficiency was the most common, followed by Factor XII, and Factor VIII deficiencies. Out of 153 patients analyzed with factor deficiencies, only one patient had FXIII deficiency making it the rarest factor deficiency along with Factor II deficiency. FXIII deficiency was also the only factor deficiency which was not seen in conjunction with any other factor deficiencies. Platelet aggregation dysfunction and thrombocytopenia were the most commonly reported platelet abnormalities [1]. Acquired von Willebrand disease due to shearing force induced by pulmonary stenosis was thought to be responsible in a few cases [7]. No correlation between genotype and bleeding disorder was noted [1].

Clinical features of FXIII deficiency range from umbilical cord bleeding in the neonatal period to intracranial hemorrhage (ICH), intramuscular or subcutaneous hematomas and prolonged bleeding post-surgery ICH is the most common cause of mortality in untreated patients [8].

Cryoprecipitate and FFP are the two most commonly used options to treat FXIII deficiency [9]. Plasma derived FXIII concentrate is approved in the United States for perioperative management and prophylaxis of congenital FXIII deficiency [10-11]. Since the plasma derived product contains both subunits A and B, it can be used to control bleeding in patients regardless of mutation in subunit A or B of FXIII [12]. Recombinant FXIII-A (rFXIIIA) subunit (catridecacog) is also approved and can only be used in patients with FXIII subunit A deficiency [13]. Prophylactic therapy is indicated in patients with moderate and severe Factor XIII deficiency (level<5) [9].

Currently, there are no validated guidelines for diagnosis of bleeding disorder in patients with NS [14]. Due to variety of bleeding diatheses and the lack of concordance between laboratory values and clinical presentation [1] diagnosis is challenging. This underscores the need of early hematologist involvement along with comprehensive laboratory testing [15]. Nugent et al. suggested that comprehensive evaluation be carried out at the diagnosis of NS, after diagnosis in the event of symptomatic patients, and before any surgical procedure, which NS patients will most likely need to undergo given frequent co-existent cardiac pathologies [1]. Also, since patients with FXIII deficiency can present with life threatening bleeding, screening for bleeding disorders in NS patients appears logical. Given lack of clear guideline, recommendations regarding screening and diagnosis of bleeding disorders in NS are needed.

## Conclusions

Noonan syndrome can be associated with any bleeding disorders (defect in platelet function or coagulation factors); the diagnosis can be challenging and broad testing looking for platelet function and factor deficiencies is needed. Given that PT/INR and PTT are initial part of bleeding disorder evaluation and can be normal in patients with FXIII deficiency, diagnosis may be missed if FXIII is not checked. Our case highlights the importance of looking for FXIII deficiency in NS.
